# Sustained Efficacy, Safety and High Adherence Rate of Onabotulinum Toxin Type A in Chronic Migraine Patients: A Multicentric Prospective Real-Life Study

**DOI:** 10.3390/toxins15010034

**Published:** 2022-12-31

**Authors:** Ilenia Corbelli, Angela Verzina, Ilaria Leone De Magistris, Gioacchino De Vanna, Paolo Eusebi, Giorgia Mataluni, Antonio Pisani, Addolorata Maria Pia Prudenzano, Maria Trojano, Marianna Delussi, Marina De Tommaso, Antonio Russo, Marcello Silvestro, Gioacchino Tedeschi, Paolo Calabresi, Paola Sarchielli

**Affiliations:** 1 Neurological Clinic, S. Maria Misericordia Hospital, Department of Medicine, University of Perugia, 06129 Perugia, Italy; 2 Neurology Unit, Department of Systems Medicine, University of Tor Vergata, 00133 Rome, Italy; 3 Headache Center, Neurological Clinic “L. Amaducci”, Department of Basic Medical Sciences, Neurosciences and Sense Organs, University of Bari, 70121 Bari, Italy; 4 Applied Neurophysiology and Pain (ANP) Unit, University of Bari Aldo Moro, 70121 Bari, Italy; 5 Department of Advanced Medical and Surgical Sciences, University of Campania “Luigi Vanvitelli”, 81100 Caserta, Italy; 6 The Institute of Neurology, Agostino Gemelli University Policlinic IRCCS, 00168 Rome, Italy; 7 Department of Neurosciences, Catholic University of the Sacred Heart, 00168 Rome, Italy

**Keywords:** chronic migraine, onabotulinumtoxinA, migraine prophylaxis

## Abstract

Guidelines regarding long-term use with onabotulinumtoxinA (onaBT-A) in chronic migraine (CM) prophylaxis are lacking. This multicentric prospective real-life study aimed to assess the efficacy and safety of a long-term treatment. A total of 195 chronic migraine patients were treated with onaBT-A, every 3 months for 5 cycles (Phase 1). In the Phase 2 of the study, depending on response rate, patients were divided into “responders” (R), “partially responders” (PR) and “non-responders” (NR). Then, we proposed to R and PR patients to continue with an additional 12 months of treatment (additional 4 sessions). Response to treatment and adverse events were collected for the entire duration of the study. Of the 195 patients included (females 82.1%, mean age 47.4 ± 12.4), at the end of Phase 1 there were 52.3% of R patients, 17.9% of PR patients, 15.4% of NR patients and 14.4% drop-outs. During Phase 2 of treatment, R patients presented a maintenance of the improvement achieved during the first year of treatment, as well as PR patients. Except for three serious adverse events not related to treatment, all other adverse events were mild or moderate in severity and resolved without sequelae. In the literature, adherence to oral migraine-preventive medications among patients with CM was found to be less than 25%. The results of this prospective real-life multicenter study show efficacy, safety and adherence to a long-term treatment with onaBT-A.

## 1. Introduction

Diagnosis of chronic migraine (CM) requires 15 or more headache days per month of which at least 8 are of migrainous type, for more than 3 months, in patients with a history of episodic migraine [1]. CM patients have great headache-related disability with low health-related quality of life [2]. The overuse of symptomatic drugs (Medication-Overuse Headache, MOH) can in many cases complicates CM, but it is also the most important risk of transformation from migraine with and/or without aura to CM [1,3].

The treatment guidelines of these conditions involve the use of various pharmacological oral preventive treatments, including antidepressants, anticonvulsants and beta-blockers, in order to reduce the frequency and the severity of attacks and to decrease the intake of acute medication [4,5,6]. However, clinical experience shows that this condition has a challenging management with refractoriness, intolerance and compliance less than 25% with oral drugs [7].

More recently, two non-oral treatments have entered the landscape of preventive therapies for migraine: the onabotulinumtoxinA (onaBT-A) and the newly approved humanized monoclonal antibodies that target calcitonin gene-related peptide (CGRP) or its receptor [4,8].

OnaBT-A is a currently approved treatment only for CM prophylaxis [4]. The registration studies showed its effectiveness and safety in the treatment of migraine of onaBT-A during one year of treatment [9,10]. There is some data about long-term treatment but guidelines regarding this condition are lacking [11,12,13].

Indeed, in a Cochrane review, Herd and Coll. stated that more data is needed to establish the long-term effect of this treatment [11]. Since then, a few studies have been published, most of them single-center, retrospective or with a small sample size [12,13,14,15,16,17,18,19,20]. Only one study was carried out in Europe but in a non-naïve population for onaBT-A [13]. In light of this, with this first Italian multicentric prospective real-life study we aimed to assess the efficacy and safety of a long-term treatment in a large sample size of adults naïve to onaBT-A CM and MOH patients.

## 2. Results

**Figure 1:**
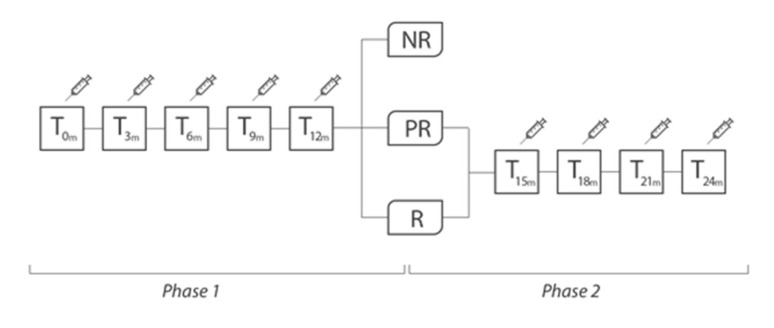
Study design. R = “responders”, patients with reduction in the number of headache days per month ≥50%); PR = “partially responders”, patients with reduction in the number of days of headache per month <50%, but ≥25%); NR = “non-responders”, patients with reduction in the number of headache days per month <25%.

**Figure 2:**
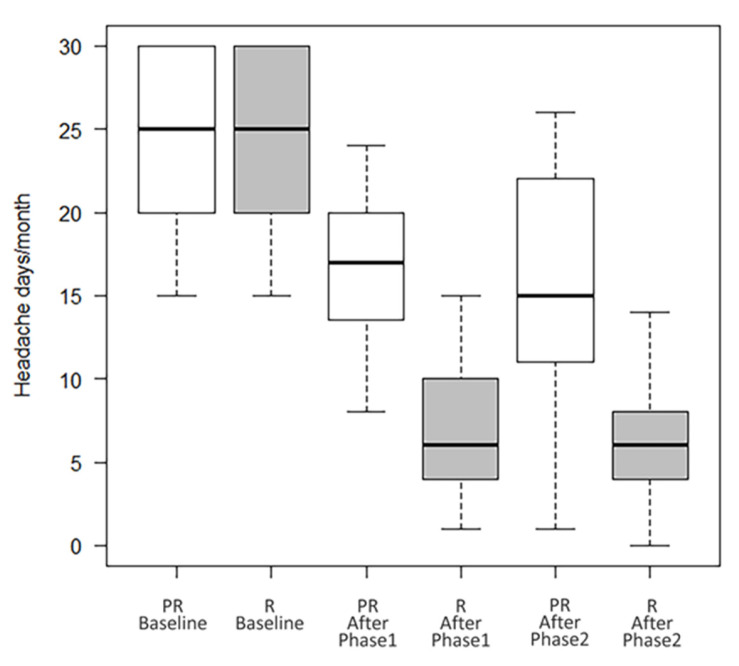
Number of headache days/month of R and PR patients at baseline, after Phase 1 and Phase 2.

Overall, 195 patients were included (females 82.1%, mean age 47.4 ± 12.4). A total of 94 patients (48.2%) were taking a concomitant oral prophylaxis and none of the enrolled patients received a new oral preventive treatment, but possibly only a modification (reduction/increase) of any therapy already in progress and only in the second year of treatment. All patients received onaBT-A 155 U spread over 31 injection sites at a dosing interval of 12 weeks in the first year of treatment. In the second year, 8 (4.1%) patients, underwent to additional 40 U over 8 injection sites according to the follow-the-pain strategy to a maximum total dose of 195 U. As shown in Table 1, at the end of the Phase 1 (see Figure 1) there were 52.3% (F/M:89/13; mean age 47.8 ± 14.2) of R patients, 17.9% (F/M:21/14; mean age 47.0 ± 12.0) of PR patients and 15.4% (F/M:24/6; mean age 45.4 ± 13.7) of NR patients. In this phase, drop-outs were 14.4% (F/M:26/2; mean age 49.5 ± 9.7). There were no statistically significant differences between the types of abused drug (paracetamol/NSAID, triptan/ergotamine, analgesics in combination, combination of analgesics) among the four groups (data not shown). During the Phase 2 of treatment, R patients maintained the improvement achieved during the first year of treatment (from 24.2 ± 5.6 headache days/month before the start of the Phase 1, to 7.0 ± 4.2 *p* < 0.001, after the Phase 1, and to 6.9 ± 5.1 at the end of the Phase 2 with *p* = ns between the values recorded at the end of the Phase 1 and those recorded at the end of the Phase 2); PR patients presented a trend toward a further improvement, although not statistically significant (from 23.8 ± 5.8 headache days/month before the start of the Phase 1, to 17.4 ± 5.3 *p* < 0.001, after the Phase 1, and to 15.3 ± 7.6 *p* = ns at the end of the Phase 2) (see Table 1 and Figure 2). In Phase 2, there were four further drop-outs. The list of reasons for withdrawal is shown in Table 2.

The list of adverse events is shown in Table 3 some of which led to discontinuation of treatment. Most adverse events are represented by feeling of contraction with cervical-brachialgia, diffuse muscle pain or localized at the injection site. Serious adverse events reported among the two phases of the study were 3: one pregnancy, one death, one hospitalization; none of them were related to onaBT-A treatment.

## 3. Discussion

In clinical practice, the management of CM patients, mostly associated with MOH, is challenging for all physicians, as these patients are more refractory to common therapeutic oral prophylaxis [21,22]. Furthermore, similar to many other chronic diseases, a common pitfall in the management of CM is the lack of compliance with treatment. In fact, less than 25% of CM patients adhere to oral migraine preventive regimen 1 year after treatment [7,23], due to a number of factors, including multi-day intake, lack of efficacy and side effects of oral prophylaxis, such as weight gain, somnolence, fatigue, hypotension [21,22].

A wide variety of medications used as preventive treatment for episodic migraine have also been used as prophylaxis therapy for CM. However, their efficacy in CM is uncertain, as few have actually been investigated in this subtype of patients. In particular, topiramate, CGRP-monoclonal antibodies and onaBT-A have been evaluated specifically as preventatives in patients with CM, while other preventive treatments, such as beta-blockers and tricyclic antidepressants, have not been sufficiently studied in these patients [11,24,25,26]. Nevertheless, the high rate of adverse events, contraindications and the potential risk of causing depression restrict topiramate use. On the other hand, CGRP-monoclonal antibodies, which were not yet available in our country at the time this study was conducted, should be considered in patients who have failed or did not tolerate traditional treatment options. Indeed, according to the European Headache Federation guidelines, CGRP-monoclonal antibodies are strongly recommended for episodic and chronic migraine prevention in light of their efficacy and safety but, due to cost and the restrictions regarding their prescribability of some countries, they are not always considered as a first-line option [26,27].

OnaBT-A has demonstrated its efficacy for the CM prophylaxis in two well-designed phase III clinical trials [9,10,11] in reducing the mean frequency of days with headache and headache episodes, compared to placebo. Since then, the use of onaBT-A has been approved by the American Food and Drug Administration and the European Medicines Agency as a second line treatment in the prophylaxis of CM.

OnaBT-A seems to be effective more than topiramate, as shown by the number of patients needed to treat (NNT) to achieve a significant reduction in the rate of migraine days, which was 8.0 and 12.5 for onaBT-A and topiramate, respectively [17]. Moreover, onaBT-A seems to have fewer treatment-related adverse effects (in the PREEMPT studies: treatment group 29.4% versus placebo 12.7%) than topiramate (treatment group 65.0% versus placebo 42.9%), which have often led to the abandonment of this latter drug [9,10,24]. Since no head-to-head trial with randomized design exists, comparing CGRP-monoclonal antibodies with onaBT-A through an adjusted indirect comparison meta-analysis, showed that they were both effective in reducing headache days with similar adverse event and tolerability rate [26].

Despite these comforting data, the duration of prophylactic treatment with onaBT-A in clinical practice remains to be fully defined. Long-term studies on efficacy and safety are therefore mandatory to understand how long the treatment has to be continued and, if effective, whether, when and for how long it has to be discontinued.

The pooled PREEMPT data showed that patients not responding to the first onaBT-A treatment cycle may well respond to the second or third. In fact, albeit half of the patients responded to the first onaBT-A treatment cycle (≥50% reduction in headache days: 49.3%), 11.3% responded to the second and other 10.3% to the third cycle, probably due to an inter-individual variability in time needed to reverse the central sensitization [28].

Although guidelines for onaBT-A treatment in CM recommend to stop treatment if no benefit is achieved in two consecutive cycles [29], it has already been shown in an observational real-life study on 56 patients that benefit and progressive conversion to responder status is achieved within 5 treatment cycles, with significant (66%) conversion rate to episodic migraine pattern [30].

Moreover, there is evidence prompting its long-term use (>1 year) in CM. In fact, onaBT-A benefits seem sustained, as documented by one study reporting that 74.2% of the 108 responders during the first year still respond to the treatment at 2 years [31]. Subsequent studies also confirmed this data [12,13,32,33,34].

The pathophysiological basis of this extremely subjective response lies in the supposed mechanisms of the action of onaBT-A. It blocks neurotransmission via Soluble N-ethylmaleimide sensitive factor Attachment protein REceptor (SNARE) complex cleavage, inhibiting the release of calcitonin gene-related peptide (CGRP), substance *p* and glutamate. Such effect, together with the regulation of the expression of the transient receptor potential vanilloid type 1, which is localized within C-fibers and participating in pain transmission, directly limit peripheral sensitization. Once peripheral sensitization is reduced, central sensitization indirectly decreased, leading to pain relief [35].

All of these factors have a role in the effect of onaBT-A on peripheral and central sensitization and can be expressed at different extents and differentially over time in individual CM patients and this might influence the treatment response [36,37,38].

In light of this, since there are no specific guidelines in this regard, a minimum of 5 administrations (12 months of treatment, 1 session every 3 months) hopefully should be carried out in clinical practice.

Furthermore, post-marketing, real-life, prospective studies confirmed the efficacy and tolerability data of the PREEMPT studies, demonstrating a reduction in headache days by >50% in 32% of patients and >75% in 14% of patients, respectively, while migraine days were reduced by >50% in 50% and >75% in 24% of cases [39].

In light of this, as recommended by the guidelines for controlled trials of prophylactic treatment of CM, in our real-life setting study, we used the classic target in reduction of headache frequency of at least 50% (R-group) but also that of the reduction of at least 25% (PR-group) [40].

As far as adverse events are concerned, a pooled analysis of five trials with multiple onaBT-A treatments (up to 5 cycles) by Diener and collaborators, showed neck pain and muscle weakness as the most common side effect, which were mild or moderate in intensity. They occurred in 72.9% of patients treated with onaBT-A and 56.8% in the placebo group, while serious adverse events were reported by 5.4% of patients receiving the active drug and 3% of patients receiving placebo [41].

Treatment-related discontinuation occurred in 7.7% of patients treated with onaBT-A and 24.1% of patients treated with topiramate [24].

In our real-life long-term multicenter study, onaBT-A was effective or partially effective in 70.3% of patients, and this efficacy was sustained over 2 years.

Our multicenter study also shows that safety and adherence rate of onaBT-A treatment in CM patients are sustained over two years of treatment. In fact, in the two years of follow-up, we detected 14.9% of adverse events of which those most probably related to the onaBT-A treatment were cervico-brachialgia (neck pain and brachialgia), pain/paresthesia and dermatitis at the injection site. Except for serious adverse events, all other adverse events were mild or moderate in severity and resolved without sequelae. Moreover, treatment-related discontinuation due to autonomous decision of suspension due to ineffectiveness or discomfort from procedure is 10.8% during the first year of treatment.

Although the pivotal trials [9,10] have tested onaBT-A up to 1 year of treatment, our real-life experience demonstrates the maintenance of efficacy, safety and adherence rate even for a prolonged treatment duration.

The major strength of our prospective study is the long-term follow-up of CM treated patients. Another strength is the multicentric setting that allowed a large sample size, which was representative of CM patients attending headache centers in our country. Conversely, there are some limitations in our study. Firstly, we used a clinically-based sample rather than a population-based sample, considering that, in our country, onaBT-A treatment is prescribed and administered only in headache centers. Moreover, the multicentric setting could have led to different management strategies (e.g., onaBT-A used as sole therapy or in add on to other oral prophylactic therapies) that may slightly differ between centers. For these reasons, selection bias cannot be excluded.

## 4. Conclusions

Most of the preventive medications used for episodic migraine have not been rigorously studied for the treatment of CM and adherence to oral migraine-preventive medications among patients with CM was found to be low. onaBT-A is the main prophylaxis approved for the treatment of CM, although its use in clinical practice remains to be fully defined with long-term studies on efficacy and safety. Our real-life multicenter study shows how the long-term efficacy and safety of onaBT-A treatment in CM patients are sustained over two years of treatment, with a high adherence rate, probably due to the method of administration.

## 5. Materials and Methods

This prospective observational “real-life” clinical study took place at five Headache Centers. It was conducted from February 2015 to October 2019, with enrollment occurring in the first 3 years of the study. Our Institutional Review Board and local Ethical Committee of every involved center approved this observational study (“CEAS Comitato Etico Aziende Sanitarie Umbria, Prot. # 3903/14/ESS", approved on: 17 September 2014).

Patients, with diagnosis of CM with or without MOH (8.2 and 1.3 ICHD-3 codes, respectively) according to the International Headache Society criteria [1], also confirmed by a 3-month diary prior to enrollment, who in clinical judgment were eligible for prophylactic treatment with onaBT-A, and were naïve to this type of treatment, were enrolled in the study. Patients were not included in the case of: (i) aged less than 18 years; (ii) administration of anesthetics or steroids in the target muscles in the 30 days prior to the start of the study, muscle relaxants (including benzodiazepines) in the 2 days before and in the 2 days following treatment with onaBT-A; (iii) concomitant diseases or medications that may expose the subject to risks with the onaBT-A administration (e.g., neuromuscular pathologies, intake of aminoglycosides, curare-like agents, or other agents that may interfere with neuromuscular function); and (iv) infections or skin diseases in the administration sites.

All included patients were treated with onaBT-A according to Phase III REsearch Evaluating Migraine Prophylaxis Therapy (PREEMPT) paradigm [9,10]. Administration of up to additional 40 UI was allowed, in line with the PREEMPT “follow the pain” paradigm, at the injector’s discretion and according to individual patients’ needs. Patients were treated with onaBT-A every 3 months for 5 cycles (Phase 1 of the study, 1 year of therapy). Then, at the beginning of the Phase 2 of the study, patients were divided into “responders” (R, patients with reduction in the number of headache days per month ≥50%), “partially responders” (PR, patients with reduction in the number of days of headache per month <50%, but ≥25%), “non-responders” (NR, patients with reduction in the number of headache days per month <25%). This subdivision was possible on the basis of clinical data recorded on headache diaries. Due to headache frequency and intake of symptomatic drugs data, after 5 onaBT-A cycles, it was possible to detect the proportion of patients who still have MOH. At the beginning of Phase 2, onaBT-A treatment was suspended for NR patients, redirecting them to other prophylaxis treatments. We proposed to R and PR patients to continue with an additional 12 months of treatment (additional 4 sessions, 1 every 3 months). R and PR patients, at the end of the Phase 2, underwent clinical follow-up visits to assess whether the efficacy of the drug, partial or complete, was sustained over time (see Figure 1 and Table 1).

Demographic and clinical data of the patients were collected. Patients were asked to fill out a daily headache diary for the entire duration of the study. They were allowed to use acute symptomatic treatment as needed. At each visit, adverse effects were recorded and then evaluated for potential relationship to onaBT-A.

The study was conducted in accordance with the principles of the Helsinki Declaration. Written informed consent for participation and publication was obtained from each patient before entering the study.

### Statistical Analysis

Categorical data were described as count and percentage, while continuous variables were summarized by means of mean and standard deviation. Statistical inference for bivariate association was performed with chi-square test for categorical data and t-test for continuous data. Repeated measurement model was used for comparing outcomes between time points. Significance level was set at *p* < 0.5. R version 3.5 was used for all the statistical analyses [42].

## Figures and Tables

**Table 1:** Clinical and demographic characteristics of included patients.

		Drop-Out Group	NR	PR	R	*p*-Value
**N (%)** **Tot = 195**		28 (14.4)	30 (15.4)	35 (17.9)	102 (52.3)	-
**N (%) MOH at Baseline**		23 (82.1)	25 (83.3)	29 (82.9)	87 (85.3)	-
**N** (**%**) **MOH at Phase 1**		-	26 (86.7)	17 (48.9)	11 (10.8)	
**N** (**%**) **MOH at Phase 2**		-	-	15 (42.9)	9 (8.8)	
**Gender** (**F/M**)		26/2	24/6	21/14	89/13	**<0.001**
**Age**		49.5 ± 9.7	45.4 ± 13.7	47.0± 12.0	47.8 ± 14.2	0.679
**Headache days/month**	**Baseline**	22.1 ± 6.6	23.7 ± 5.4	23.8 ± 5.8	24.2 ± 5.6	0.375
	**Phase 1**	-	22.7 ± 6.7 ϕϕϕ	17.4 ± 5.3 §§§	7.0 ± 4.3 §§§	**<0.001**
	**Phase 2**	-	-	15.3 ± 7.6 ***	6.9 ± 5.1 ***	**<0.001**

NR: “non-responders”, patients with reduction in the number of headache days per month < 25%; PR: “partially responders”, patients with reduction in the number of days of headache per month < 50%, but ≥25%; R: “responders”, patients with reduction in the number of headache days per month ≥50); *p*-value column refers to a comparison between groups (drop-outs, NR, PR, R); ɸɸɸ *p* = ns intra-group comparison (NR between Baseline and the end of Phase 1; §§§: *p* < 0.001 intra-group comparison (R or PR) between Baseline and the end of Phase 1; ***: *p* = ns intra-group comparison (R or PR) between the end of Phase 1 and the end of Phase 2.

**Table 2:** Reasons of discontinuation of onabotulinumtoxinA treatment (*n* = 32).

	During Phase 1 N (%)	During Phase 2 N (%)
**Serious adverse events**	3 (9.4)	0
**Lost to follow-up/transfer abroad**	4 (12.5)	4 (12.5)
**Autonomous decision of suspension due to ineffectiveness/discomfort for procedure**	21 (65.6)	0

**Table 3:** List of adverse events of onabotulinumtoxinA treatment and probability of correlation to treatment.

	N/29	Correlation to Treatment
**Cervical-brachialgia**	6	Probable
**Flu-like syndrome**	4	Improbable
**Abdominal pain**	4	Improbable
**Diffuse muscles pain**	3	Possible
**Internal tremor**	2	Improbable
**Pain/paresthesia at the injection site**	2	Probable
**Pregnancy**	1	Not related
**Death**	1	Not related
**Hospitalization**	1	Not related
**Hypertensive crisis**	1	Improbable
**Allergic rhinitis**	1	Improbable
**Shingles**	1	Improbable
**Lower limb oedema**	1	Improbable
**Injection site dermatitis**	1	Probable

## Data Availability

The data that support the findings of this study are available on request from the corresponding author.
